# Exercise rehabilitation for patients with critical illness: a randomized controlled trial with 12 months of follow-up

**DOI:** 10.1186/cc12835

**Published:** 2013-07-24

**Authors:** Linda Denehy, Elizabeth H Skinner, Lara Edbrooke, Kimberley Haines, Stephen Warrillow, Graeme Hawthorne, Karla Gough, Steven Vander Hoorn, Meg E Morris, Sue Berney

**Affiliations:** 1 Department of Physiotherapy, The University of Melbourne, Melbourne, Australia; 2 Physiotherapy Department, Austin Health, Melbourne, Australia; 3 Department of Intensive Care, Austin Health, Melbourne, Australia; 4 Department of Psychiatry, The University of Melbourne, Melbourne, Australia; 5 Cancer Nursing Research Centre, Peter MacCallum Cancer Institute, Melbourne, Australia; 6 Department of Mathematics and Statistics, The University of Melbourne, Melbourne, Australia; 7 School of Allied Health, Latrobe University Melbourne, Melbourne, Australia

**Keywords:** Critical illness, Exercise, Health-related quality of life, Physical function, Physiotherapy, Rehabilitation

## Abstract

**Introduction:**

The purpose of this trial was to investigate the effectiveness of an exercise rehabilitation program commencing during ICU admission and continuing into the outpatient setting compared with usual care on physical function and health-related quality of life in ICU survivors.

**Methods:**

We conducted a single-center, assessor-blinded, randomized controlled trial. One hundred and fifty participants were stratified and randomized to receive usual care or intervention if they were in the ICU for 5 days or more and had no permanent neurological insult. The intervention group received intensive exercises in the ICU and the ward and as outpatients. Participants were assessed at recruitment, ICU admission, hospital discharge and at 3-, 6- and 12-month follow-up. Physical function was evaluated using the Six-Minute Walk Test (6MWT) (primary outcome), the Timed Up and Go Test and the Physical Function in ICU Test. Patient-reported outcomes were measured using the Short Form 36 Health Survey, version 2 (SF-36v2) and Assessment of Quality of Life (AQoL) Instrument. Data were analyzed using mixed models.

**Results:**

The *a priori* enrollment goal was not reached. There were no between-group differences in demographic and hospital data, including acuity and length of acute hospital stay (LOS) (Acute Physiology and Chronic Health Evaluation II score: 21 vs 19; hospital LOS: 20 vs 24 days). No significant differences were found for the primary outcome of 6MWT or any other outcomes at 12 months after ICU discharge. However, exploratory analyses showed the rate of change over time and mean between-group differences in 6MWT from first assessment were greater in the intervention group.

**Conclusions:**

Further research examining the trajectory of improvement with rehabilitation is warranted in this population.

**Trial registration:**

The trial was registered with the Australian New Zealand Clinical Trials Registry ACTRN12605000776606.

## Introduction

Each year in Australia, more than 125,000 patients are admitted to the ICU, of whom more than 80% survive [1]. In England, this figure is nearly 240,000 [2]. There is growing evidence that many survivors experience long-term physical, neurocognitive and mental health complications directly associated with their ICU experience. This has been termed post–intensive care syndrome (PICS) [3].

The provision of exercise rehabilitation has been advocated to address the weakness and functional limitation observed in ICU survivors [4-6]. Despite this, only a few controlled intervention trials have quantified its effectiveness beyond hospital discharge [7-9], with one community-based trial providing follow-up to 6 months [10]. No study has yet reported the effects of providing rehabilitation as a continuum from inpatient to outpatient care and measured outcomes at 12 months after ICU discharge. To the best of our knowledge, our present study is the first trial documenting Australian rehabilitation outcomes that include within-hospital intervention with 12-month follow-up.

The primary purpose of this study was to assess the effectiveness of an intensive physiotherapy exercise rehabilitation program commencing at day 5 after ICU admission, progressing daily on the acute hospital ward and being administered twice weekly for 8 weeks in the outpatient setting, compared with usual care, on physical function as measured using the Six-Minute Walk Test (6MWT) at 12 months after ICU discharge. Secondary aims were to assess differences in function using the Timed Up and Go (TUG) Test, Physical Function in ICU Test (PFIT) and health-related quality of life (HRQoL) at 12 months using both the Assessment of Quality of Life (AQoL) Instrument and the Short Form 36 Health Survey, version 2 (SF-36v2). A detailed study protocol has previously been published and is available online [11]. The specific details of the physiotherapy intervention have been published elsewhere [12], and the results were previously reported in abstract form [13].

## Materials and methods

Human Research Ethics approval was obtained from Austin Health, Melbourne, Australia. Informed consent was obtained from the patients or their substitute decision-makers prior to enrollment, and the trial was registered with the Australian New Zealand Clinical Trials Registry (ACTRN12605000776606). The conduct and reporting of the trial conforms to CONSORT extension guidelines [14].

We conducted a single-center, stratified, phase II, randomized controlled parallel group trial with assessor blinding in a 20-bed tertiary ICU in Melbourne, Australia. The hypothesis for the primary aim was that, compared with patients who receive usual care, patients who underwent intensive rehabilitation in ICU, hospital and community settings would demonstrate greater improvement in physical function as measured using the 6MWT at 12 months. To be eligible for enrollment, adult participants had to reside within a 50-km radius of the hospital; to have no neurological, spinal or musculoskeletal dysfunction preventing participation in physical rehabilitation; and to have an ICU length of stay (LOS) of at least 5 days. An ICU LOS more than 5 days was deemed to represent a prolonged ICU stay. This was defined as double the average LOS for ICU survivors in the previous 18-month period (2.5 days) at Austin Health, Melbourne (Australian and New Zealand Intensive Care Society (ANZICS) portal [15]. Individual participation was agreed upon by the attending intensivist. Within strata, participants were randomly assigned to receive either usual care plus exercise rehabilitation or usual care alone. An independent statistician performed the randomization by creation of a random numbers table and use of color-coded (for stratification), opaque, numbered envelopes. Physiotherapists other than those who provided usual care performed the trial intervention across the trial continuum. The outcome assessor was blinded to group allocation, and the success of blinding was measured with a short questionnaire at each time point. The strata were diagnosis classification (medical or surgical) and mechanical ventilation at day 5 (yes or no), creating four possible groups.

### Usual care

In both groups, physiotherapists provided both respiratory and mobility management based upon individual patient assessment [12] according to unit protocols. Administration of intravenous sedation in the ICU was titrated to achieve a Richmond Agitation Sedation Scale score between −1 and +1 [16] for each patient. In the usual-care arm, mobility may have included active bed exercises, sitting out of bed and/or marching or walking. Usual care was available 7 days per week for 12 hours per day. Acute ward physiotherapy services emphasized functional recovery and discharge planning. Outpatient exercise classes for ICU survivors were not included in usual-care physiotherapy at the hospital.

### Intervention arm

**Figure 1:**
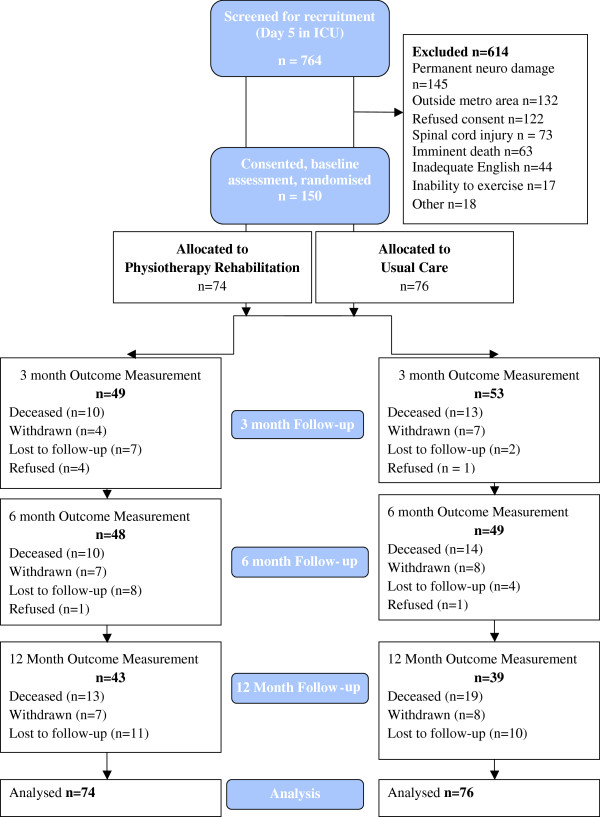
Participant flow through the trial.

Intervention was individualized based upon participant level and results of baseline physical function tests [17]. The criteria for safety and ceasing the intervention were set *a priori* and published previously in the protocol paper [11]. An overview of intervention in ICU, on the acute ward and in outpatients is provided in Table 1 and the Additional file 1. The intervention was designed to provide more active functional rehabilitation based upon physiological principles of exercise prescription, in all phases of the study than would be received as part of usual care. The timing of outcomes post hospital discharge are given in Figure 1.

**Table 1:** **Exercise rehabilitation in ICU, ward and outpatient settings**^**a**^

	**ICU**		**Ward**	**Outpatients**
Frequency of exercise sessions	Mechanically ventilated	Weaned		
	15 min/day	2 × 15 min/day	2 × 30 min/day progressed to 1 × 60 min/day	60 min twice weekly for 8 wk
Type of exercise	Marching in place		Cardiovascular, progressive resistance strength training and functional exercise	Cardiovascular, progressive resistance strength training and functional exercise
	Moving from sitting to standing			
	Arm and leg active and active resistance movements			
Protocol breach	<10 min/session		<20 min/session	
Repetitions	Prescribed from baseline PFIT and Modified Borg Scale		Prescribed from baseline 6MWT, cycle ergometer, 5RM	Prescribed from pre-outpatient 6MWT, cycle ergometer, 5RM
Intensity	Target Modified Borg Scale score 3 to 5		Modified Borg Scale score 4 to 6	Modified Borg Scale score 4 to 6
			Commenced at 70% peak walking speed	Commenced at 70% peak walking speed

^a^5RM, repetition maximum used for strength training; PFIT, Physical Function in the ICU Test; 6MWT, Six-Minute Walk Test. *Weaned* means successfully weaned from mechanical ventilation or able to be disconnected from mechanical ventilation for more than 4 hours/day. Weaning from mechanical ventilation was achieved using a standardized unit weaning protocol.

### Statistical analyses

The study was designed to enroll 200 patients to provide a statistical power of 80% to detect a mean difference in 6MWT at 6 months of 50 m using a standard deviation of 110 m, including allowance for loss to follow-up [18]. All descriptive data were analyzed using SPSS for Windows version 18.0 software (SPSS, Chicago, IL, USA). Analyses of the outcome data were performed using SAS for Windows version 9.3 software (SAS Institute, Inc, Cary, NC, USA). The primary outcome (6MWT) was analyzed on the basis of a linear mixed model with group (usual care or intervention) and time (treated as categorical with levels at ICU discharge, hospital discharge, and 3, 6 and 12 months post–ICU discharge). Linear mixed models use all data available at each time point; thus missing data imputation was not undertaken. Stratification factors (diagnosis classification: medical or surgical, mechanical ventilation at day 5 yes or no) were also included as covariates by adding them to the regression model. A similar approach was used for the secondary outcomes (TUG, AQoL and SF-36) and applied to all available data. Analyses were pragmatic and based on the intention-to-treat principle, which included data on all randomized participants with at least one outcome measure. Analysis of covariance was used to examine the group difference for PFIT with adjustment for the stratification factors as well as the baseline measure of PFIT. The exploratory analysis also used a linear mixed model, but with time considered as a continuous term in the model. This allowed the investigation of the rate of change between groups.

Descriptive data analysis, individual change scores and 95% confidence intervals were calculated between first assessment (in ICU for PFIT, prerandomization for AQoL and SF-36v2 and at ICU discharge or ward arrival for 6MWT and TUG) and follow-up assessments at ICU discharge for PFIT and at 3 and 12 months using observed data for the remaining measures. These values were compared with the minimal clinically important difference for each outcome. Differences in ICU-acquired weakness (ICUAW), measured at day 7 postawakening, between those who were ventilated at day 5 and those who were not were examined using independent *t*-tests. Further details of statistical analyses are available in Additional file 1.

## Results

Participants were recruited from May 2007 until August 2009, with follow-up completed by September 2010. Participant flow through the trial is described in Figure 1. The predetermined sample size goal of 200 was not achieved. Individuals who consented to participate represented a cohort of medical and surgical patients with a moderate illness severity score and a mean age of 60 years who had been admitted to the ICU for 5 days or more (Table 2). Only 8% were never intubated, and 55% were still mechanically ventilated (MV) on day 5 after admission, with a median length of MV of 4 days. Medical diagnoses made up 82 patients (55%) in the sample population, and 112 (75%) had at least one chronic disease comorbidity. The acute hospital readmission rate was 41% in both groups at 12 months. Thirty-four participants (23%) were referred for inpatient rehabilitation, and this was evenly distributed between groups. Of the subgroup (stratum) that included participants who remained ventilated at day 5 post–ICU admission, 20 (38%) of 53 met criteria for ICUAW compared with 9 (17%) of 53 of the participants who were not ventilated at day 5 post–ICU admission, and this difference was significant (*P* = 0.017). However, there were no significant between-group differences in the incidence of ICUAW (defined as Medical Research Council score less than 48 of 60) (Table 2). There were no other significant between-group differences in any of the patient variables.

**Table 2:** **Characteristics of trial participants**^**a**^

**Variable**	**Usual care**	**Intervention**
	**(*n*** **= 76)**	**(*n*** **= 74)**
Mean age (SD) (yr)	60.1 (15.8)	61.4 (15.9)
Gender (% male)	68.4	58.1
Mean BMI (SD)	27.7 (6.1)	27.5 (5.4)
Mean APACHE II score (SD)	20.7 (7.7)	19 (6)
ICU diagnosis (%)		
Pneumonia	17	17
Cardiac	12	11
Cardiac surgery	22	23
Other surgery	16	15
Liver disease/transplant	7	14
Cardiac arrest	8	3
Sepsis	7	10
Renal	4	3
Other	7	4
Chronic disease (%)	74	76
Respiratory	39	23
Cardiac	53	53
Diabetes	30	26
Arthritides	9	9
More than one chronic disease	42	28
28-day mortality (%)	7.9	8.1
12-month mortality (%)	25.0	17.6
ICU LOS (days), median (IQR)	7.0 (6.0 – 11.0)	8.0 (6.0 – 12.0)
ICU LOS ≥10 days (%)	32.0	40.5
Acute LOS (days), median (IQR)	20.0 (13.0-30.8)	23.5 (16.0 - 41.5)
ICUAW (% yes)	17.1	21.6
MV hours, median (IQR)	98.0 (47.5-160.5)	105.0 (52.0-216.5)
MV at day 5, % (n)	55.3 (42)	55.4 (41)
Readmissions, % (n)	40.8 (31)	41.9 (31)
Discharge location		
Home % (n)	52.6 (40)	59.5 (44)
Rehabilitation	25.0 (19)	20.3 (15)
Acute hospital	5.3 (4)	5.4 (4)
Other	17.1 (13)	14.9 (11)

^a^APACHE II, Acute Physiology and Chronic Health Evaluation II; BMI, body mass index; ICU LOS, intensive care unit length of stay; ICUAW, intensive care unit–acquired weakness, measured at 7 days after awakening; IQR, interquartile range; MV, mechanical ventilation; SD, standard deviation. Readmissions are defined as acute hospital readmission during the 12-month trial follow-up period. Discharge locations are defined as rehabilitation = inpatient rehabilitation, acute hospital = transferred to another acute hospital and other = palliative care, transitional care or deceased.

There were no major adverse events during exercise intervention according to our *a priori* safety criteria [11], and overall mortality at 12 months was 32 participants (21%). A further 36 participants (24%) had withdrawn or were lost to follow-up at 12 months, 20 (13%) by the 3-month time point.

Details of blinding and compliance with assessments (Additional files 2 and 3) are presented in Additional file 1. Once initiated, the treatment intervention was unable to be completed in the ICU on 5% of the occasions and on 6% of occasions on the ward. Although no individual session was unable to be completed in the outpatient setting, 44 (72%) of 61 participants commenced rehabilitation and 25 (41%) of 61 completed the classes (more than 70% attendance required). Ten participants were deceased and three withdrew from the trial prior to commencement of the outpatient program. There were no statistically significant differences in the demographics of the 59% who did not complete outpatient assessments compared with those who did complete outpatient assessments (see Additional file 4) or the rest of the sample. Further information related to outcomes for outpatient noncompleters and completers can be found in Additional file 4.

The raw descriptive data (Table 3) demonstrate that both the usual-care and intervention groups improved in all outcomes up to 12 months. There was a high level of variability in results, as demonstrated by the size of the standard deviations presented in Table 3. Compared with population norms, the 6MWT, TUG, AQoL and SF-36 results at 12 months remained below predicted values for age and gender. The 6MWT and AQoL scores were 60% to 65% of Australian norm-based scores at 12 months [19,20].

**Table 3:** **Six-Minute Walk Test, Timed Up and Go Test, Physical Function in ICU Test, Assessment of Quality of Life Instrument utility and Short Form 36 Health Survey, version 2 raw scores by study group**^**a**^

	**Measurement time point**												
		**Baseline**		**ICU discharge**		**Discharge to home**		**3 months post–ICU discharge**		**6 months post–ICU discharge**		**12 months post–ICU discharge**	
**Outcome measure**		***n***	**Mean (SD)**	***n***	**Mean (SD)**	***n***	**Mean (SD)**	***n***	**Mean (SD)**	***n***	**Mean (SD)**	***n***	**Mean (SD)**
6MWT (m)	Usual care			60	187.9 (126.1)	58	266.7 (136.8)	52	382.1 (139.4)	45	402.4 (166.6)	38	409.6 (158.5)
	Intervention			63	146.4 (79.4)	59	244.2 (124.0)	48	384.5 (147.9)	44	394.2 (156.2)	41	433.8 (150.7)
TUG (s)	Usual care			60	36.1 (42.9)	57	12.9 (6.6)	53	11.6 (11.2)	48	12.9 (17.9)	40	14.2 (24.7)
	Intervention			63	41.1 (43.2)	59	18.8 (24.5)	51	12.2 (10.0)	47	9.8 (5.1)	45	10.3 (6.2)
PFIT	Usual care	72	5.2 (3.0)	56	8.0 (1.5)								
	Intervention	72	5.1 (3.1)	61	7.7 (1.7)								
AQoL (utility)	Usual care	56	0.6 (0.3)					66	0.5 (0.4)	63	0.6 (0.4)	57	0.5 (0.4)
	Intervention	52	0.6 (0.3)					59	0.5 (0.4)	58	0.5 (0.4)	56	0.5 (0.4)
SF-36v2 PF	Usual care	56	42.8 (13.1)					52	42.3 (12.0)	48	42.4 (13.7)	38	44.0 (11.2)
	Intervention	52	39.6 (15.3)					49	39.9 (14.4)	48	40.1 (14.7)	42	41.4 (12.5)
PCS	Usual care	56	41.7 (11.5)					51	42.1 (9.6)	47	44.4 (10.7)	38	46.2 (9.4)
	Intervention	51	39.3 (12.9)					49	41.0 (11.4)	47	41.6 (13.2)	42	44.7 (10.9)
MCS	Usual care	56	44.3 (12.8)					51	46.3 (12.0)	47	46.2 (12.9)	38	44.7 (15.7)
	Intervention	51	41.8 (13.3)					49	46.0 (13.9)	47	45.8 (12.9)	42	47.9 (12.3)

^a^6MWT, Six-Minute Walk Test (meters), higher scores equal improved performance, normative value = 662 m; TUG, Timed Up and Go Test (seconds), lower scores equal improved performance, normative value = 9.19 s; PFIT, Physical Function in ICU Test, scored on the basis of Rasch analysis, interval score range 0 to 10, with higher scores indicating improved performance; AQoL, Assessment of Quality of Life Measure; consistent with utility theory, for those who died, the AQoL utility score was set at 0.00 and higher scores indicate higher utility; SF-36v2, Short Form 36 Health Survey, version 2, in which higher scores indicate improved performance; PF, SF-36v2 physical function domain; PCS, SF-36v2 physical component score, MCS, SF-36v2 mental component score. SF-36v2 results are presented as T-scores where the population mean is 50 and the SD is 10. With the SF-36v2, only survivors at each time point contribute data. Baseline AQoL and SF-36 were administered to participants as close as possible to enrollment in the trial as a “then test” whereby participants retrospectively estimated their premorbid health-related quality of life.

### Physical function performance

**Figure 2:**
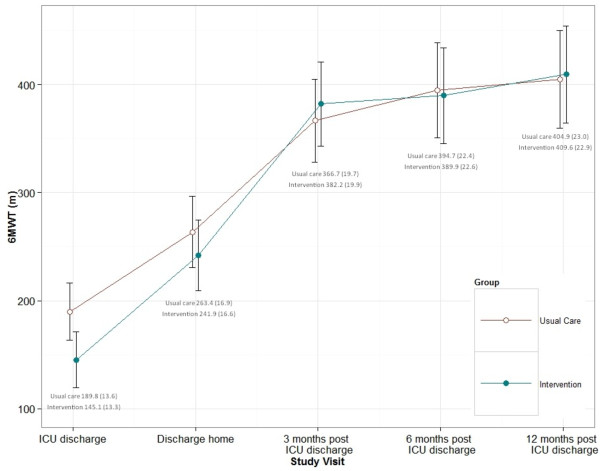
**Mean Six-Minute Walk Test (standard error) by study group calculated from model estimates.** Note that the calculated population mean for this sample (ages 60 to 69 years) is 539 m. The steeper incline of the intervention group line, particularly between discharge to home and 3-month post–ICU discharge study visit, indicates a greater rate of improvement in Six-Minute Walk Test (6MWT) distance.

There was a difference in 6MWT at the first measure (ICU discharge). The intervention group walked a significantly shorter distance, but there were no significant differences at any subsequent time point, including at 12 months, based upon the model estimates (Table 4). The data for the primary outcome measure, 6MWT, are presented in Figure 2. There were no significant differences between the groups in TUG improvements at any time point. Adjusting for the four *a priori* subgroups (ventilated or not ventilated at day 5 plus either medical or surgical condition) did not change outcomes measured using the 6MWT or TUG (results not shown). There was no difference between the intervention and usual-care groups within the ICU in function measured using the PFIT with mean differences between groups of −0.3 (95% CI −0.9 to 0.3, *P* = 0.343).

**Table 4:** **Group comparisons for Six-Minute Walk Test from the model estimates**^**a**^

**Study visit**	**Usual care**	**Intervention**	**Mean difference from usual care**
	**mean 6MWT (SE)**	**mean 6MWT (SE)**	**(95% CI, *P *** **value)**
ICU discharge	189.8 (13.6)	145.1 (13.3)	−44.7 (−82.3 to −7.1, 0.020)
Discharge to home	263.4 (16.9)	241.9 (16.6)	−21.5 (−68.4 to 25.4, 0.365)
3 months post–ICU discharge	366.7 (19.7)	382.2 (19.9)	15.4 (−40.1 to 71, 0.583)
6 months post–ICU discharge	394.7 (22.4)	389.9 (22.6)	−4.9 (−68.0 to 58.3, 0.879)
12 months post–ICU discharge	404.9 (23.0)	409.6 (22.9)	4.7 (−59.7 to 69.2, 0.884)

^a^6MWT, Six-Minute Walk Test; SE, standard error; CI, confidence interval. Means were calculated and comparisons between groups were made at each time point from the linear mixed model. Subject numbers are the same as those reported in Table 3. Values given are in meters.

Although there were no differences between groups at any time point on the 6MWT, the steeper upward slope to 3 months of the intervention group shown in Figure 2 demonstrated that the trajectory of recovery may be different for the intervention and control arms of the study; therefore, we undertook exploratory analyses to describe these differences over the trial period. Group was a significant predictor of rate of change (group × time; *P* = 0.049), with the intervention group showing greater improvement compared with the usual-care group (Table 5). Between-group differences in mean changes from first assessment to 3 months and 12 months were moderate (effect size = 0.53 and 0.54, respectively) and greater than the reported minimal clinically important difference (MCID) (see Additional file 1), although the confidence intervals were wide (Table 6) [21,22]. There were no between-group differences in TUG rate of change (*P* = 0.072), and these differences were less than the reported MCID (Table 5).

**Table 5:** **Rate-of-change results for Six-Minute Walk Test and Timed Up and Go Test**^**a**^

**Parameter**	**6MWT estimate (95% CI)**	**TUG estimate (95% CI)**
Group	−29.60	4.41
	(−67.32 to 8.11)	(−2.53 to 11.35)
Time	12.12	−1.23
	(10.05 to 14.20)**	(−1.62 to −0.85)**
Quad time	−0.16	0.020
	(−0.20 to −0.13)**	(0.013 to 0.027)**
Group × time	2.95	−0.20
	(0.016 to 5.88)**	(−0.41 to 0.018)
Group × quad time	−0.044	
	(−0.098 to 0.010)	

^a^6MWT, Six-Minute Walk Test; TUG, Timed Up and Go Test. Time represents the average number of weeks since first assessment (ICU discharge or ward arrival). Variance components are available from the authors. Time represents the change over time from first assessment in both groups. A positive value represents an increased score (6MWT distance increase – improved function), negative values represent decreased scores (TUG time decrease – improved function). Quad time represents the rate of change between time points of measurement. Group × time represents the treatment effect in this analysis: the interaction between group and time. Compared to the control group, the intervention group 6MWT distance improved by an average of 2.95 m/wk. Group × quad time represents the interaction between rate of change and group. **Significant results.

**Table 6:** **Outcome measure mean change from first assessment and effect size**^**a**^

		**Usual care**			**Intervention**			**Difference**		
**Outcome measure**	**Time point**	***n***	**Mean change**	ES*	***n***	**Mean change**	**ES**	**Difference**	**95% CI**	ES^**^
6MWT (m)	3 months	49	184.25	1.67	47	247.93	3.59	63.67	14.17 to 113.18^+^	0.53
	12 months	35	219.49	1.98	40	292.04	4.40	72.55	9.29 to 135.81^+^	0.54
TUG (s)	3 months	51	−23.37	0.55	50	−31.68	0.72	−8.31	−24.90 to 8.28	0.20
	12 months	38	−19.52	0.47	44	−29.09	0.69	−9.57	−27.42 to 8.28	0.24
AQoL utility	3 months	53	−0.08	0.27	43	0.037	0.15	0.12	−0.03 to 0.26	0.33
	12 months	48	−0.11	0.36	40	0.031	0.12	0.14	–-.03 to 0.31	0.36
SF-36v2 PF	3 months	45	−3.2	0.24	39	3.64	0.24	6.8	1.2 to 12.5^+^	0.53
	12 months	35	0.39	0.03	33	3.88	0.27	3.5	−3.5 to 10.5	0.25
PCS	3 months	45	−2.26	0.19	38	3.33	0.26	5.6	0.09 to 11.1^+^	0.45
	12 months	35	3.33	0.28	32	6.46	0.49	3.1	−3.2 to 9.5	0.25
MCS	3 months	44	3.12	0.24	38	5.56	0.41	2.4	−3.6 to 8.5	0.18
	12 months	35	2.59	0.19	32	7.50	0.57	4.9	−2.7 to 12.5	0.32

^a^6MWT, Six-Minute Walk Test distance (meters); AQoL, Assessment of Quality of Life Measure score; CI, confidence interval: ^+^significant differences between groups occur when CI does not cross 0; ES, effect size; MCS, SF-36v2 Mental Component score; PCS, SF-36v2 Physical Component score; PF, SF-36v2 Physical Function domain; SF-36v2, Short Form 36 Health Survey, version 2; TUG, Timed Up and Go Test (seconds). *ES, effect size = mean change from first assessment/standard deviation at first assessment. A positive mean change reflects improvement at follow-up assessment relative to baseline (participants with missing assessments excluded). **ES, effect size = (intervention mean change – usual care mean change)/pooled standard deviation for change. A positive difference reflects intervention advantage in terms of overall improvement.

### Patient-reported outcomes: health-related quality of life

There were no between-group differences in AQoL utility scores at any time point (Additional file 5), and no domains of the SF-36v2 demonstrated between-group differences at any time point (Additional files 5, 6 and 7). Adjusting for *a priori* subgroups (ventilated at day 5 and medical or surgical) did not change outcomes for HRQoL measures. Sensitivity analyses involving identification and removal of outliers did not markedly alter the results presented herein (data not shown).

## Discussion

Our research presented herein is the first to assess the effectiveness of a continuum of physiotherapy-led rehabilitation from the ICU through outpatient rehabilitation with 12-month follow-up in a mixed medical and surgical population in Australia. Primary outcomes analysis based upon differences in 6MWT results showed no significant differences between study groups at 12 months. Neither study arm returned to a functional exercise capacity level equivalent to population norms. However, exploratory analyses demonstrated an increased rate of improvement in 6MWT results for the intervention group and differences greater than MCID from first assessment to 3- and 12-month follow-up. There were no differences found for HRQoL or other secondary outcomes.

Our patient characteristics compare well with those described in other work undertaken with general ICU patients [8,9]. Their conditions were less acute than some [7], and they were older than many of the patients with acute lung injury reported elsewhere [23,24]. With results similar to those of the current study, Elliott *et al*. did not find significant differences in 6MWT or HRQoL at 8 or 26 weeks after discharge in a similar population (APACHE II (20 vs 20), mechanical ventilation hours (101 vs 83 h) and age (60 v 57 yr)) [10]. This trial intervention consisted solely of an outpatient home-based program. Previous randomized controlled trials (RCTs) conducted in the United States [9] and Belgium [7] reported beneficial outcomes at hospital discharge from therapy-led rehabilitation; however, no previous RCT followed up participants for 12 months. The HRQoL domains of the SF-36v2 remained lower than population norms up to 12 months, again consistent with other trials and longitudinal follow-up data, and physical function domains were worst affected [10,24-26].

Rehabilitation standards of care vary internationally. This is illustrated well when comparing the results of our study to those of others [9,27,28]. Our usual care involved physiotherapy 7 days per week for 12 hours per day and included early mobilization practices (sitting out of bed, marching on the spot), but not mobilization away from the bed while ventilated. Standard care for 68 patients at our center reported that 52% of patients mobilized in the ICU [12]. This is more usual care physiotherapy than reported in North American studies, where only 12.5% of patients received any physical therapy in the ICU in the study by Morris and colleagues [27]. Pohlman and colleagues reported that 63% of participants in their intervention arm, who were not mechanically ventilated, were mobilized in the ICU; but none received mobility exercises while ventilated in their usual-care group [9,29]. Although our usual care did not include walking away from the bed during MV, as yet there is no evidence that this achieves improved outcomes compared with marching on the spot next to the bed or other functional mobility exercises. These differences in usual care practices may contribute to the lack of separation between our groups compared with others [9,27]. Furthermore, examining outcomes after hospital discharge demonstrates that 53% of patients in the usual-care group and 59% in the intervention group were discharged to home in our trial. This compares with 24% of usual-care patients and 43% of intervention patients in the study by Schweickert *et al*. [9]. This clearly highlights that, despite similar patient demographics in the two trials, a higher percentage of patients in both of our groups went home. Also, they may reflect differing health system practices between countries where referral systems and nurse- and physiotherapist-to-patient ratios differ [30], leading to difficulty in comparing data internationally. European physiotherapy models are more similar to those of Australasia [31], but to date only one trial from Belgium has been reported [7], and it recruited patients with higher acuity and longer ICU stay with follow-up to hospital discharge.

The 12-month 6MWT values reached only 60% to 65% of normal population values and were consistent with the findings of others [23,25]. Given that we are unable to measure premorbid 6MWT and that many ICU survivors have chronic disease in addition to their presenting diagnoses [23], it is likely that their premorbid function starts lower than population norms and therefore remains low at follow-up. Because of this factor, it may be preferable in the future to include age and comorbid disease as stratification variables. As this area of clinical interest has developed in the past 6 years, the influence of these factors is clearer today than in 2007, when we designed our trial and determined our strata.

We decided *a priori* to measure differences in 6MWT at each time point [11]; however, evaluating the gains in 6MWT over time from the ICU may be a preferable hypothesis. The fact that both groups improved from baseline in most outcome measures suggests that our exploratory analyses of measuring the rate of improvement in 6MWT and the mean differences gained across time in each group provide interesting and valuable information about the trajectory and timing of improvement. Figure 2 and Table 3 demonstrate the comparative improvement in the intervention group between discharge to home and the 3-month time point in 6MWT. The intervention group 6MWT started lower, which we hypothesize may be the result of increased MV hours, LOS and diagnosis of ICUAW being somewhat worse in this group (Table 2). Although these data alone were not individually significant, they may together represent a sicker population. The effects of providing exercise over this time frame of recovery can be cumulative [32]. Furthermore, achievement of the MCID in an outcome such as the 6MWT can be compared using this design. Issues in calculating and applying the MCID have been highlighted [33], but this measure does allow a clinical benefit to be compared rather than relying solely on inferential analyses. The 6MWT MCID has not yet been published specifically for survivors of critical care. Without more detailed evidence defining the trajectory of physical function recovery, it is difficult to hypothesize about the shape and slope of the recovery graph over time. If we believe this to be a smooth recovery toward the premorbid slope as described by Iwashyna’s “big hit” theory [34], then the triple-phase exercise intervention we applied in this study resulted in the treatment group reaching their peak recovery more quickly. This has implications for return to the highest levels of function and immersion in daily activities, including return to work. It may also be relevant to the timing of study functional impairment end points in future research.

What are the lessons to be drawn from this trial? This patient group is a difficult, heterogeneous population to study. Figure 1 attests to this, with high numbers of patients who were unable to be recruited. In most situations, next of kin were approached first, and consent was difficult to obtain because their consideration for their sick loved ones was paramount; thus making decisions on their behalf regarding exercise and research was difficult [35]. The failure to reach our enrollment targets makes our negative results difficult to interpret. The fact that we had available only data at ICU discharge for sample calculations in 2007 contributed to this problem. The variability in our primary outcome of 6MWT at hospital discharge was 125 m, but at 12 months it was 153 m. This variability increases the sample needed for separation at 12 months. Our outpatient attendance was 41%, raising questions whether our lack of response was a result of inadequate delivery of the intervention. Although we did not include specific qualitative analyses, we hypothesize that the sample heterogeneity, including age and comorbid disease, may have affected outpatient physiotherapy attendance. Younger participants and those without a sepsis diagnosis (Additional file 4) were unable or unwilling to return to outpatient classes. Had we designed a more flexible outpatient program that could be performed locally in a community setting or in the home rather than one that required a return to hospital outpatients, delivery of the intervention may have improved. Additionally, targeting only those participants with impairments on repeat testing may have yielded different results. Although similar to attendance at outpatient rehabilitation by patients with chronic obstructive pulmonary disease [36-39], we did not provide maintenance exercise support up to 12 months as recommended in pulmonary rehabilitation guidelines [40]. Given the number of participants with persistent functional impairments at 12 months, this may have improved responses to our intervention. It is possible that the combination of high levels of usual-care physiotherapy, sample size and inability to provide the intervention in all participants or for only those with impairments contributed to lack of significant between-group differences. Furthermore, restricting recruitment to a more homogeneous population as defined by age groups, comorbidities, sepsis and diagnosis groups may reduce sample size requirements in future trials. Given the results reported by Burtin *et al*. [7], it is also possible that patients with a longer ICU stay may benefit more from exercise intervention, although this needs to be tested with inclusion of longer follow-up measures. Indeed, the subgroup of patients still ventilated at day 5 after ICU admission had a more than twofold increase in diagnosis of ICUAW in our study. It may be these patients who need to be targeted for early rehabilitation. However, more evidence for the direct relationship between muscle strength and functional impairment is warranted. We did not test for delirium or any cognitive or psychological sequelae of ICU admission. Recent research has demonstrated these to be common and important impairments in ICU survivors [41], and more research combining physical and cognitive interventions in rehabilitation is warranted [42].

Recent research highlights the importance of functional outcomes to ICU survivors [43,44]; however, the type, quantity, timing and length of rehabilitation intervention needed are not yet established [45]. The variability across time in the 6MWT highlights a need for identification of more sensitive and specific objective outcome measures or a “package” of measures to be developed in this population. More than one measure may be required to measure different activity limitations and disability at different time points of recovery. Using HRQoL measures that report on participation may not be interchangeable with those measuring activity restriction, such as performance-based tests like the 6MWT [46]. Although our trial was well-conducted, adhered to protocol, attempted long-term follow-up, maintained (single) blinding and scrutinized all available data using intention-to-treat and linear mixed models, it was a single-center study, which limits the generalizability of our findings.

Future research that examines very early rehabilitation in the ICU, such as cycling exercise [12], that seeks to identify patients at risk for worse muscle weakness and the benefits of including more ward-based intervention [47] are warranted. Furthermore, studies that include measurements of participant self-efficacy and resilience as well as caregiver attributes and support will also be important, as these factors may impact outcomes. Indeed, it is possible that these less tangible and unmeasured factors may have influenced between-group differences in our study.

## Conclusion

In this single-center RCT measuring therapist-led exercise rehabilitation in three phases from ICU though to outpatient classes, physical function recovery as measured by the 6MWT at 12 months was not different between usual-care and intervention cohorts. Furthermore, HRQoL was not different between groups at any time point after randomization.

## Key messages

• This single-center RCT shows that physical function measured using the 6MWT was not significantly different between intervention and usual-care groups at 12-month follow-up in an Australian ICU population.

• HRQoL was not significantly different between groups.

• The rate of improvement for the 6MWT was significantly better in the intervention group based on exploratory analysis. This outcome warrants further investigation.

• The data presented in this paper will be useful for the design of future trials in this area.

## Abbreviations

5RM: Five-repetition maximum; 6MWT: Six-Minute Walk Test; ACTRN: Australian New Zealand Clinical Trials Registry Number; ANZICS: Australian and New Zealand Intensive Care Society; APACHE II: Acute Physiology and Chronic Health Evaluation; AQoL: Assessment of Quality of Life; BMI: Body mass index; CI: Confidence interval; HRQoL: Health-related Quality of Life; ICU: Intensive care unit; ICUAW: Intensive care unit-acquired weakness; IQR: Interquartile range; LOS: Length of stay; MCID: Minimal clinically important difference; MCS: Mental component score on the SF-36v2; MV: Mechanical ventilation; PCS: Physical component score on the SF-36v2; PF: Physical function domain on the SF-36v2; PFIT: Physical Function in ICU Test; PICS: Post–intensive care syndrome; SD: Standard deviation; SE: Standard error; SF-36v2: Short Form 36 version 2; TUG: Timed Up and Go Test.

## Competing interests

The authors declare that they have no competing interests.

## Authors’ contributions

LD was involved in the trial conception and design and in trial management. ES was involved in the trial conception and design and in data collection. LE participated in the data monitoring and trial management. KH participated in the data collection. GH was involved in the trial conception and design. KG and SVH performed the data analyses. SW was involved in the trial management. MM was involved in data monitoring. SB was involved in the trial conception and design and in trial management. All authors were involved in writing and revising the manuscript. All authors read and approved the final manuscript.

## Supplementary Material

Additional file 1 On-line supplement information, additional intervention, statistical analyses and result information. Click here for file

Additional file 2: Table S1 Compliance with questionnaires/assessments. Click here for file

Additional file 3: Table S2 Reason for non-compliance. Click here for file

Additional file 4: Table S3 Demographics and outcomes of intervention outpatient non-attenders and attenders. Click here for file

Additional file 5: Table S4 Group comparisons for secondary outcomes from the model estimates. Click here for file

Additional file 6: Table S5 Additional SF-36v2 raw domain scores mean (SD) by study group. Click here for file

Additional file 7: Table S6 Group comparisons for additional SF-36 domain scores from model estimates. Click here for file
