# Supplementary material for: Exercise rehabilitation for patients with critical illness: a randomized controlled trial with 12 months of follow-up
Source: Crit Care. 2013 Jul 24;17(4):R156. doi: 10.1186/cc12835 (PMC4056792; doi:10.1186/cc12835)
Supplement: Additional file 1 — On-line supplement information, additional intervention, statistical analyses and result information. [file cc12835-S1.docx]

On-line supplement information.

**Intervention arm**

Intervention was individualized based upon participant level and results of baseline physical function tests. ^E^[^1^](#_ENREF_1) The intervention was provided by five physiotherapists (post graduate experience ranging from 4-6 years) for six days per week across the trial period. The criteria for safety and ceasing the intervention were set apriori and published previously in the protocol paper. ^E^[^2^](#_ENREF_2) An overview of intervention in ICU, on the acute ward and in out patients is provided in Table 1. The intervention was designed to provide more active functional rehabilitation based upon physiological principles of exercise prescription, in all phases of the study than would be received as part of usual care. We prescribed the exercises using a physiological (reproducible) approach rather than basing these on subjective therapist assessments. We used the baseline functional tests (six minute walk test (6MWT) and physical function in ICU test (PFIT-s) ^E^[^3^](#_ENREF_3) to set parameters to commence exercising and progress exercises during rehabilitation. Outpatient group classes were commenced an average (SD) of 11 (13) days after hospital discharge. Completion was defined as attending >70 % (>11 sessions) of the 16 sessions available. ^E^[^4^](#_ENREF_4) One intervention physiotherapist and an exercise physiologist supervised the outpatient program. A home walking program was prescribed but not formally monitored. Complete details of the intervention are previously published. ^E^[^5^](#_ENREF_5) The timing of outcomes post ICU discharge are given in Figure 1.

**Statistical Analyses**

The study was designed to enroll 200 patients to provide a statistical power of 80% to detect a mean difference in 6MWT at six months of 50m using a standard deviation of 110m, including allowance for loss to follow up. ^E^[^6^](#_ENREF_6) All descriptive data were analyzed using SPSS Windows Version 18.0 (SPSS, Chicago, IL, USA), including baseline data, compliance with questionnaire and functional assessments as well as reasons for non-compliance by study arm. Analyses of the outcome data were performed using SAS software for Windows Version 9.3 (SAS Institute, Inc., Cary, NC). The primary outcome (6MWT) was analysed with a linear mixed model with group (usual care or intervention) and time (treated as categorical with levels ICU discharge, hospital discharge, and 3 months, 6 months, and 12 months post-ICU discharge). Linear mixed models use all data available at each time point and thus missing data imputation was not undertaken. Stratification factors (diagnosis classification: medical or surgical, mechanical ventilation at day 5: yes/no) were also included as covariates by adding to the regression model. Results from each analysis are presented as group means, as well as the difference between groups, together with 95% confidence intervals at each time point as determined using the mixed model. A similar approach was used for the secondary outcomes (TUG, AQoL, and SF36) and applied to all available data. Appropriate covariance structure for analyses of all primary and secondary outcomes was formally examined using a likelihood ratio test. Analyses were pragmatic and based on the intention-to-treat principle which included data on all randomized participants with at least one outcome measure. Analyses for all primary and secondary outcomes were fitted with an unstructured covariance structure in the linear mixed model. Analysis of covariance was used to examine the group difference for PFIT-s with adjustment for the stratification factors as well as the baseline measure of PFIT-s. Additional analyses were carried out to assess the robustness of estimated group differences to obvious outliers and/or influential data points.

The exploratory analysis also used a linear mixed model but with time considered as a continuous term in the model. This allowed the investigation of the rate of change between groups. Fully parameterized models included fixed effects for time (linear and quadratic components) and group (standard care, standard care plus rehabilitation). Quadratic terms were removed if tests were marginal (p > 0.2). In this model Quadtime represents the rate of change between time points of measurement; Group*time represents the treatment effect which is the interaction between group and time; Group*Quadtime represents the interaction between rate of change and group.

Descriptive data analysis, individual change scores and 95% confidence intervals were calculated between first assessment (in ICU for PFIT-s; pre-randomization for AQoL and SF36v2; at ICU discharge/ward arrival for 6MWT and TUG) and follow-up assessments at ICU discharge for PFIT-s and 3 and 12 months using observed data for remaining measures. The within-group effect size was calculated as the mean change from first assessment divided by the standard deviation at first assessment. The between-group effect size was calculated as the difference between study arms in mean change from first assessment divided by the pooled standard deviation of change. ^E^[^7^](#_ENREF_7)^,^[^8^](#_ENREF_8) These values were compared with the minimal clinically important difference for each outcome. Values used were: 35m for 6MWT, ^E^[^9^](#_ENREF_9) 9.5 seconds for TUG, ^E^[^10^](#_ENREF_10) 0.06 utilities for the AQoL utility score, ^E^[^11^](#_ENREF_11) and 5 points for the SF 36v2. ^E^[^12^](#_ENREF_12) Differences in intensive care unit acquired weakness (ICUAW), between those who were ventilated at day 5 and those who were not, were examined using independent t-tests.

**Results**

Compliance with assessments

There were no differences in compliance with outcome assessments (see Additional file 2) or reasons for non-compliance (see Additional file 3). The rate of outcome assessor un-blinding was 11/150 (7%); these were all intervention participants. On subsequent occasions, outcome assessments were performed by a different blinded assessor.

References

**E****1.** Skinner EH, Berney S, Warrillow S, Denehy L. Development of a physical function outcome measure (PFIT) and a pilot exercise training protocol for use in intensive care. *Critical Care and Resusitation.* 2009;11:110-115.

**E2.** Denehy L, Berney S, Skinner E, et al. Evaluation of Exercise Rehabilitation for Survivors of Intensive Care: Protocol for a Single Blind Randomised Controlled Trial. *The Open Critical Care Medicine Journal.* 2008;1:39-47. <http://www.benthamscience.com/open/toccmj/articles/V001/39TOCCMJ.pdf>.

**E3.** Denehy L, Skinner EH, Edbrooke L, et al. A Physical Function Test for use in the ICU: Validity, responsiveness and predictive utility of the PFIT (scored). *American Thoracic Society.* Vol 183. Denver: American Review of Respiratory and Critical Care Medicine; 2011:1001 - 6477.

**E4.** Keating A, Lee A, Holland AE. What prevents people with chronic obstructive pulmonary disease from attending pulmonary rehabilitation? A systematic review. *Chron Respir Dis.* 2011;8(2):89-99.

**E5.** Berney S, Haines K, Skinner E, Denehy L. An exercise prescription approach to rehabilitation for survivors of critical illness. *Physical Therapy Journal.* 2012;92:1524-1535.

**E6.** Burtin C, Clerckx B, Robbeets C, et al. Effectiveness of early exercise in critically ill patients: preliminary results. *Intensive Care Medicine.* 2006; 32(109).

**E7.** Kazis L, Anderson J, Meenan R. Effect sizes for interpreting changes in health status. *Medical Care.* 1989;27:S178-189.

**E8.** Yancy W, Almirall D, Maciejewski M. Effects of two weight-loss diets on health-related quality of life. *Qual Life Res.* 2009;18:281-289.

**E9.** Puhan M, Scharplatz M, Troosters T, Walters EH, Steurer J. Pulmonary rehabilitation following exacerbations of chronic obstructive pulmonary disease (Review). *The Cochrane Library.* 2009(1):1-31.

**E10.** de Morton NA, Keating JL, Jeffs K. Exercise for acutely hospitalised older medical patients. *Cochrane Database Syst Rev.* 2007(1):CD005955.

**E11.** Hawthorne G, Osborne R. Population Norms and Meaningful Differences for the Assessment of Quality of Life (AQoL) Measure. *Australia and New Zealand Journal of Public Health.* 2005;29(7):136-142.

**E12.** Ware J, Kosinski M, Dewey J. *How to score Version 2 of the SF-36 Health Survey*. Lincoln: Quality Metric Inc; 2000.
