# Supplementary material for: Exercise rehabilitation for patients with critical illness: a randomized controlled trial with 12 months of follow-up
Source: Crit Care. 2013 Jul 24;17(4):R156. doi: 10.1186/cc12835 (PMC4056792; doi:10.1186/cc12835)
Supplement: Additional file 2: Table S1 — Compliance with questionnaires/assessments. [file cc12835-S2.docx]

Table E1. Compliance with questionnaires / assessments

| **Time point** | **Outcome measure** | **Control** | | **Intervention** | |
| --- | --- | --- | --- | --- | --- |
|  |  | **(*N*=76)** | | **(*N*=74)** | |
|  |  | **No. received / assessed** | **% compliance** | **No. received / assessed** | **% compliance** |
| **Recruitment** | SF36v2 | 56 | 73.7 | 52 | 70.3 |
|  | AQoL | 56 | 73.7 | 52 | 70.3 |
| **ICU discharge / ward arrival** | 6MWT | 60 | 78.9 | 63 | 85.1 |
|  | TUG | 60 | 78.9 | 63 | 85.1 |
| **Hospital discharge** | 6MWT | 58 | 76.3 | 59 | 79.7 |
|  | TUG | 57 | 75.0 | 59 | 79.7 |
| **3 months post ICU discharge** | 6MWT | 52 | 68.4 | 48 | 64.9 |
|  | TUG | 53 | 69.7 | 51 | 68.9 |
|  | SF36v2 | 52 | 68.4 | 49 | 66.2 |
|  | AQoL | 53 | 69.7 | 49 | 66.2 |
| **6 months post-ICU discharge** | 6MWT | 45 | 59.2 | 44 | 59.5 |
|  | TUG | 48 | 63.2 | 47 | 63.5 |
|  | SF36v2 | 48 | 63.2 | 48 | 64.9 |
|  | AQoL | 49 | 64.5 | 48 | 64.9 |
| **12 months post-ICU discharge** | 6MWT | 38 | 50.0 | 41 | 55.4 |
|  | TUG | 40 | 52.6 | 45 | 60.8 |
|  | SF36v2 | 38 | 50.0 | 42 | 56.8 |
|  | AQoL | 39 | 51.3 | 43 | 58.1 |

Footnotes

SF36v2 = Short Form 36 Version 2; AQoL = Assessment of Quality of Life measure. Analysis of AQoL data includes patients lost to follow up due to death, patients who die are assigned a utility score of '0'; 6MWT = six minute walk test; TUG = timed up and go test. Compliance rates are presented for the total sample, including those who had deceased prior to completion of the study.
