# Supplementary material for: Exercise rehabilitation for patients with critical illness: a randomized controlled trial with 12 months of follow-up
Source: Crit Care. 2013 Jul 24;17(4):R156. doi: 10.1186/cc12835 (PMC4056792; doi:10.1186/cc12835)
Supplement: Additional file 3: Table S2 — Reason for non-compliance. [file cc12835-S3.docx]

Table E2. Reason for non-compliance

| **Time point** | **Outcome measure** | **Reason** | **Control** | **Intervention** |
| --- | --- | --- | --- | --- |
|  |  |  | **(n=76)** | **(n=74)** |
|  |  |  | **No.** | **No.** |
| **Recruitment** | SF36v2/AQoL | withdrawn | 2 | 0 |
|  |  | patient confused | 17 | 20 |
|  |  | patient unable to complete | 1 | 1 |
|  |  | patient unable to recall | 0 | 1 |
| **ICU discharge / ward arrival** | 6MWT/TUG | deceased | 7 | 7 |
|  |  | withdrawn | 7 | 2 |
|  |  | missed assessment | 2 | 2 |
| **Hospital discharge** | 6MWT/TUG | deceased | 9 | 10 |
|  |  | withdrawn | 7 | 3 |
|  |  | missed assessment | 2 | 1 |
|  |  | refused | 0 | 1 |
| **3 months post-ICU discharge** | 6MWT | deceased | 13 | 10 |
|  |  | withdrawn | 7 | 4 |
|  |  | missed assessment | 3 | 6 |
|  |  | refused | 1 | 2 |
|  |  | unreachable | 0 | 2 |
|  |  | measures taken in the home | 0 | 2 |
|  | TUG | deceased | 13 | 10 |
|  |  | withdrawn | 7 | 4 |
|  |  | missed assessment | 2 | 5 |
|  |  | refused | 1 | 2 |
|  |  | unreachable | 0 | 2 |
|  | SF36v2/AQoL | deceased | 13 | 10 |
|  |  | withdrawn | 7 | 4 |
|  |  | missed assessment | 2 | 5 |
|  |  | refused | 1 | 3 |
|  |  | unreachable | 0 | 2 |
|  |  | patient confused | 0 | 1 |
| **6 months post-ICU discharge** | 6MWT | deceased | 14 | 10 |
|  |  | withdrawn | 8 | 7 |
|  |  | missed assessment | 4 | 3 |
|  |  | refused | 3 | 4 |
|  |  | unreachable | 1 | 4 |
|  |  | measures taken in the home | 1 | 2 |
|  | TUG | deceased | 14 | 10 |
|  |  | withdrawn | 8 | 7 |
|  |  | missed assessment | 3 | 2 |
|  |  | refused | 2 | 4 |
|  |  | unreachable | 1 | 4 |
|  | SF36v2/AQoL | deceased | 14 | 10 |
|  |  | withdrawn | 8 | 7 |
|  |  | missed assessment | 3 | 3 |
|  |  | refused | 1 | 1 |
|  |  | unreachable | 1 | 5 |
| **12 months post-ICU discharge** | 6MWT/TUG | deceased | 19 | 13 |
|  |  | withdrawn | 8 | 7 |
|  |  | missed assessment | 1 | 0 |
|  |  | refused | 1 | 2 |
|  |  | unreachable | 8 | 8 |
|  |  | measures taken in the home | 1 | 1 |
|  |  | deceased | 19 | 13 |
|  |  | withdrawn | 8 | 7 |
|  |  | missed assessment | 0 | 2 |
|  |  | refused | 1 | 0 |
|  |  | unreachable | 8 | 7 |
|  | SF36v2/AQoL | deceased | 19 | 13 |
|  |  | withdrawn | 8 | 7 |
|  |  | missed assessment | 1 | 3 |
|  |  | unreachable | 9 | 8 |

Footnotes

SF36v2 = Short Form 36 Version 2; AQoL = Assessment of Quality of Life measure. Analysis of AQoL data includes patients lost to follow up due to death; patients who die are assigned a utility score of '0'; 6MWT = six minute walk test; TUG = timed up and go test.
