# Supplementary material for: Exercise rehabilitation for patients with critical illness: a randomized controlled trial with 12 months of follow-up
Source: Crit Care. 2013 Jul 24;17(4):R156. doi: 10.1186/cc12835 (PMC4056792; doi:10.1186/cc12835)
Supplement: Additional file 4: Table S3 — Demographics and outcomes of intervention outpatient non-attenders and attenders. [file cc12835-S4.docx]

Table E3. Demographics and outcomes of intervention outpatient non-attenders and attenders.

|  | Outpatient non-attender | | | Outpatient attender | | |
| --- | --- | --- | --- | --- | --- | --- |
| **Demographics** | n | Median | IQR | n | Median | IQR |
| Age (mean, SD) | 36 | 57.06 | 17.01 | 25 | 62.72 | 14.46 |
| BMI (mean, SD) | 36 | 28.15 | 5.79 | 22 | 28.39 | 4.78 |
| APACHE II | 36 | 18.00 | 14.25-21.50 | 25 | 17.00 | 15.50-20.00 |
| ICU LOS | 36 | 7.00 | 5-10 | 25 | 7.00 | 6-13 |
| Acute LOS | 36 | 24.50 | 12-40.25 | 25 | 23.00 | 16-34.50 |
| MV hours | 32 | 67.92 | 26.63-212.83 | 24 | 109.50 | 45.63-185.13 |
| Male n (%) | 36 | 20/36 (56%) |  | 25 | 15/25 (60%) |  |
| Septic (yes) n(%) | 36 | 5/36 (14%) |  | 25 | 8/25 (32%) |  |
| Vent day 5 n (%) | 36 | 17/36 (47%) |  | 25 | 15/25 (60%) |  |
| ICUAW n (%) | 29 | 6/29 (21%) |  | 19 | 5/19 (26%) |  |
| ICU re-admission 12/12 n (%) | 36 | 6/36 (17%) |  | 25 | 7/25 (28%) |  |
| Hospital re-admission 12/12 n (%) | 36 | 19/36 (53%) |  | 25 | 10/25 (40%) |  |
| **Outcomes** |  |  |  |  |  |  |
| 6MWT 3 months (mean, SD) | 24 | 380.00 | 160.71 | 24 | 388.94 | 137.29 |
| 6 months (mean, SD) | 20 | 390.10 | 196.16 | 24 | 397.58 | 117.51 |
| 12 months | 17 | 453.00 | 298.50-515.00 | 24 | 420.00 | 359.25-500.50 |
| TUG 3 months | 27 | 8.53 | 6.87-16.83 | 24 | 9.09 | 6.87-11.33 |
| 6 months | 22 | 7.71 | 6.57-13.62 | 25 | 8.43 | 6.86-10.47 |
| 12 months | 20 | 7.90 | 6.62-12.58 | 25 | 8.81 | 6.82-10.98 |
| SF36v2 PF 3 months | 26 | 43.30 | 20.43-52.45 | 23 | 47.88 | 31.87-52.45 |
| 6 months | 24 | 45.59 | 19.86-54.74 | 24 | 46.73 | 28.44-50.17 |
| 12 months | 18 | 47.88 | 25.00-53.02 | 24 | 44.45 | 34.15-50.17 |
| SF36v2 PCS 3 months | 26 | 41.40 | 26.23-49.89 | 23 | 45.66 | 35.74-52.13 |
| 6 months | 23 | 47.28 | 23.18-54.06 | 24 | 44.91 | 35.03-53.63 |
| 12 months | 18 | 48.31 | 33.73-55.15 | 24 | 47.33 | 38.62-50.28 |
| SF36v2 MCS 3 months | 26 | 47.66 | 42.04-57.53 | 23 | 49.33 | 38.89-56.83 |
| 6 months | 23 | 48.93 | 34.17-56.86 | 24 | 49.91 | 30.37-55.26 |
| 12 months | 18 | 51.12 | 25.80-54.28 | 24 | 53.84 | 41.25-58.79 |
| AQoL 3 months | 26 | 0.73 | .34-.92 | 23 | 0.78 | .49-.89 |
| 6 months | 24 | 0.65 | .15-.96 | 24 | 0.70 | .41-.87 |
| 12 months | 22 | 0.84 | .32-.94 | 24 | 0.75 | .66-.93 |

Footnotes

BMI = body mass index; APACHE II = acute physiology and chronic health evaluation, version II; ICU LOS = intensive care unit length of stay; MV = mechanical ventilation; Vent day 5 = ventilated on day 5 of ICU admission; ICUAW = intensive care unit acquired weakness; Re-admissions = re-admission during the 12 month trial follow up period; 6MWT = six minute walk test distance (meters); TUG = timed up and go test (seconds); SF36v2 = Short Form 36 Version 2; PF = physical function domain; PCS = physical component score; MCS = mental component score; AQoL = Assessment of quality of life score.
