# Supplementary material for: Exercise rehabilitation for patients with critical illness: a randomized controlled trial with 12 months of follow-up
Source: Crit Care. 2013 Jul 24;17(4):R156. doi: 10.1186/cc12835 (PMC4056792; doi:10.1186/cc12835)
Supplement: Additional file 5: Table S4 — Group comparisons for secondary outcomes from the model estimates. [file cc12835-S5.docx]

| **Outcome** | **Mean difference from usual care at each individual time point**  **(95% CI; p-value)** | | | | |
| --- | --- | --- | --- | --- | --- |
|  | ICU discharge | Discharge home | 3 months post ICU discharge | 6 months post ICU discharge | 12 months post ICU discharge |
| TUG  (s) | 5.2  (-10.1 to 20.5;0.503) | 5.6  (-1 to 12.2; 0.098) | -1.8  (-8.3 to 4.7; 0.585) | -2.0  (-6.9 to 2.8; 0.410) | -7.3  (-18.9 to 4.4; 0.217) |
| AQoL  (utility) | -- | -- | 0.0  (-0.1 to 0.1; 0.970) | -0.1  (-0.2 to 0.1; 0.366) | 0.0  (-0.1 to 0.2; 0.746) |
| SF36  (PF) | -- | -- | 3.4  (-1.4 to 8.2; 0.164) | 3.2  (-2.2 to 8.5; 0.240) | 1.6  (-3.7 to 7; 0.541) |
| SF36  (PCS) | -- | -- | 2.9  (-1.2 to 6.9; 0.163) | 0.6  (-4 to 5.1; 0.808) | 0.3  (-4.3 to 4.8; 0.901) |
| SF36  (MCS) | -- | -- | 1.2  (-3.7 to 6; 0.628) | 1.2  (-4.2 to 6.7; 0.651) | 5  (-1.1 to 11.1; 0.108) |

Table E4: Group comparisons for secondary outcomes from the model estimates

Footnotes

Means and comparisons between groups were made at each time point from the linear mixed model. Subject numbers are the same as those reported in table 3. TUG = Timed Up and Go Test, positive value indicates intervention group performed worse than usual care group; AQoL = Assessment of Quality of Life measure utility score, positive value indicates intervention group performed better than usual care group; SF36v2 = Short Form Health Survey Version 2, positive value indicates intervention group performed better than usual care group; PF = Physical function domain; PCS = Physical component score; MCS = Mental component score; CI = Confidence interval
