# Supplementary material for: Exercise rehabilitation for patients with critical illness: a randomized controlled trial with 12 months of follow-up
Source: Crit Care. 2013 Jul 24;17(4):R156. doi: 10.1186/cc12835 (PMC4056792; doi:10.1186/cc12835)
Supplement: Additional file 6: Table S5 — Additional SF-36v2 raw domain scores mean (SD) by study group. [file cc12835-S6.docx]

Table E5. Additional SF36v2 raw domain scores mean (SD) by study group

| **Outcome** | **Measurement time point** | | | | | | | | |
| --- | --- | --- | --- | --- | --- | --- | --- | --- | --- |
| **SF36v2**^*^ |  | Baseline  (then-test) | | 3 months post ICU discharge | | 6 months post ICU discharge | | 12 months post ICU discharge | |
|  |  | n | Mean (SD) | n | Mean (SD) | n | Mean (SD) | n | Mean (SD) |
| **Role physical** | Usual care | 56 | 40.39 (13.38) | 52 | 38.94 (11.69) | 49 | 43.92 (11.57) | 39 | 44.79 (11.62) |
|  | Intervention | 52 | 37.60 (14.32) | 49 | 39.86 (14.30) | 48 | 41.02 (13.38) | 43 | 44.23 (10.55) |
| **Bodily pain** | Usual care | 56 | 46.01 (14.89) | 53 | 49.20 (12.31) | 49 | 49.94 (14.27) | 39 | 51.89 (11.59) |
|  | Intervention | 52 | 44.46 (16.71) | 49 | 46.63 (13.86) | 48 | 46.63 (15.68) | 43 | 51.15 (12.79) |
| **General health** | Usual care | 56 | 41.64 (10.09) | 53 | 42.71 (10.93) | 48 | 43.57 (10.89) | 39 | 42.55 (11.34) |
|  | Intervention | 52 | 38.58 (10.92) | 49 | 41.71 (12.22) | 48 | 41.90 (13.48) | 43 | 42.97 (11.95) |
| **Vitality** | Usual care | 56 | 40.52 (13.26) | 53 | 41.93 (10.76) | 49 | 44.10 (11.60) | 39 | 44.89 (13.27) |
|  | Intervention | 52 | 37.20 (12.59) | 49 | 43.74 (13.30) | 47 | 43.12 (14.35) | 43 | 46.33 (12.69) |
| **Social function** | Usual care | 56 | 40.59 (15.24) | 53 | 43.51 (14.24) | 49 | 46.13 (13.23) | 39 | 44.84 (15.49) |
|  | Intervention | 52 | 38.96 (14.99) | 49 | 43.39 (15.42) | 48 | 42.19 (14.79) | 43 | 47.98 (12.89) |
| **Role emotion** | Usual care | 56 | 46.64 (14.00) | 53 | 48.07 (11.03) | 49 | 46.84 (12.87) | 39 | 44.91 (16.51) |
|  | Intervention | 52 | 42.35 (17.00) | 49 | 43.63 (16.22) | 48 | 44.49 (14.18) | 43 | 45.28 (13.55) |
| **Mental health** | Usual care | 56 | 43.01 (12.48) | 53 | 44.74 (14.02) | 49 | 45.55 (12.96) | 39 | 45.86 (16.47) |
|  | Intervention | 51 | 41.49 (13.86) | 49 | 45.67 (14.15) | 48 | 44.30 (14.82) | 43 | 46.69 (13.11) |

Footnotes

SF36v2 = Short Form 36 Version 2.
