# Supplementary material for: Exercise rehabilitation for patients with critical illness: a randomized controlled trial with 12 months of follow-up
Source: Crit Care. 2013 Jul 24;17(4):R156. doi: 10.1186/cc12835 (PMC4056792; doi:10.1186/cc12835)
Supplement: Additional file 7: Table S6 — Group comparisons for additional SF-36 domain scores from model estimates. [file cc12835-S7.docx]

Table E6: Group comparisons for additional SF36 domain scores from model estimates

| **SF36 Domain** | **Mean difference from usual care**  **(95% CI; p-value)** | | |
| --- | --- | --- | --- |
|  | 3 months post ICU discharge | 6 months post ICU discharge | 12 months post ICU discharge |
| Role physical | 4.8  (-0.6 to 10.1; 0.079) | 0.3  (-4.5 to 5; 0.917) | 1.1  (-4.0 to 6.2; 0.669) |
| Bodily pain | -0.3  (-5.5 to 4.9; 0.908) | -1.9  (-8.0 to 4.3; 0.546) | -1.2  (-6.6 to 4.2; 0.658) |
| General health | 2.1  (-2.0 to 6.2; 0.311) | 0.9  (-3.9 to 5.6; 0.718) | 3.2  (-1.6 to 8.1; 0.189) |
| Vitality | 5.4  (0.6 to 10.2; 0.029) | 2.7  (-2.7 to 8; 0.323) | 5.5  (0.2 to 10.8; 0.040) |
| Social function | 2.4  (-3.8 to 8.7; 0.442) | -2.9  (-9.1 to 3.2; 0.349) | 2.1  (-4.5 to 8.6; 0.532) |
| Role emotion | -0.6  (-5.2 to 3.9; 0.783) | -1.5  (-7.2 to 4.2; 0.608) | 4.0  (-2.7 to 10.7; 0.235) |
| Mental health | 1.9  (-3.2 to 7.0; 0.463) | 3.0  (-2.2 to 8.1; 0.254) | 3.5  (-2.6 to 9.6; 0.255) |

Footnotes

Means and comparisons between groups were made at each time point from the linear mixed model.

CI = confidence interval
